# Mechanical Ventilation and Clinical Outcomes in Patients with Acute Myocardial Infarction: A Retrospective Observational Study

**DOI:** 10.1371/journal.pone.0151302

**Published:** 2016-03-15

**Authors:** Antonio Eduardo P. Pesaro, Marcelo Katz, Jason N. Katz, Carmen Sílvia Valente Barbas, Marcia R. Makdisse, Alessandra G. Correa, Marcelo Franken, Carolina Pereira, Carlos V. Serrano, Renato D. Lopes

**Affiliations:** 1 Hospital Israelita Albert Einstein, São Paulo, SP, Brazil; 2 Division of Cardiology, University of North Carolina at Chapel Hill, Chapel Hill, North Carolina, United States of America; 3 Pulmonary Department, Heart Institute (INCOR), University of São Paulo Medical School, São Paulo, SP, Brazil; 4 Heart Institute (INCOR), University of Sao Paulo Medical School, São Paulo, SP, Brazil; 5 Duke University Medical Center, Duke Clinical Research Institute, Durham, North Carolina, United States of America; 6 Federal University of São Paulo, Paulista School of Medicine, São Paulo, SP, Brazil; University of Louisville, UNITED STATES

## Abstract

**Purpose:**

Patients with acute myocardial infarction (AMI) and respiratory impairment may be treated with either invasive or non-invasive mechanical ventilation (MV). However, there has been little testing of non-invasive MV in the setting of AMI. Our objective was to evaluate the incidence and associated clinical outcomes of patients with AMI who were treated with non-invasive or invasive MV.

**Methods:**

This was a retrospective observational study in which consecutive patients with AMI (n = 1610) were enrolled. The association between exclusively non-invasive MV, invasive MV and outcomes was assessed by multivariable models.

**Results:**

Mechanical ventilation was used in 293 patients (54% invasive and 46% exclusively non-invasive). In-hospital mortality rates for patients without MV, with exclusively non-invasive MV, and with invasive MV were 4.0%, 8.8%, and 39.5%, respectively (*P*<0.001). The median lengths of hospital stay were 6 (5.8–6.2), 13 (11.2–4.7), and 28 (18.0–37.9) days, respectively (*P*<0.001). Exclusively non-invasive MV was not associated with in-hospital death (adjusted HR = 0.90, 95% CI 0.40–1.99, *P* = 0.79). Invasive MV was strongly associated with a higher risk of in-hospital death (adjusted HR = 3.07, 95% CI 1.79–5.26, *P*<0.001).

**Conclusions:**

In AMI setting, 18% of the patients required MV. Almost half of these patients were treated with exclusively non-invasive strategies with a favorable prognosis, while patients who needed to be treated invasively had a three-fold increase in the risk of death. Future prospective randomized trials are needed to compare the effectiveness of invasive and non-invasive MV for the initial approach of respiratory failure in AMI patients.

## Introduction

Approximately 17% of patients with acute myocardial infarction (AMI) typically experience respiratory impairment due to decompensated heart failure [1]. Depending on the severity of the respiratory impairment, patients may be treated with either invasive or non-invasive mechanical ventilation (MV) [2,3]. Prior studies have shown that nearly 8% of patients with AMI are treated with invasive MV during their hospital stay [3], forming a very high-risk subgroup with almost 50% short-term mortality rates [4].

On the other hand, several trials and meta-analyses have demonstrated that non-invasive MV may be useful for the treatment of cardiogenic pulmonary edema [5,6]. Both continuous positive airway pressure and bi-level positive airway pressure ventilation, by improving gas exchange and hemodynamics, have been proven effective and safe in reducing the need for endotracheal intubation [7]. Unfortunately, most of these studies were not performed in acutely ischemic patients, and only small trials have tested non-invasive MV in the setting of AMI [8,9].

Given the limitations of the contemporary literature, the objective of our study was to assess the incidence and associated clinical outcomes of unselected AMI patients with respiratory failure requiring non-invasive or invasive MV.

## Materials and Methods

### Population

This was a retrospective observational study in which 1610 consecutive patients with ST-segment elevation myocardial infarction (STEMI) and non-STEMI were enrolled between 2004 and 2012.

AMI was defined according to international guideline criteria [10]: typical rise and gradual fall of biochemical markers of myocardial necrosis (troponin or creatine kinase-MB) with at least one of the following: 1) ischemic symptoms, 2) development of pathologic Q waves on the electrocardiogram (ECG), 3) ECG changes indicative of ischemia (ST-segment elevation or depression), or 4) coronary artery intervention (e.g., coronary angioplasty). The registry design, methods, and main results have been reported previously [11].

Clinical characteristics and in-hospital outcomes were compared among three groups of patients: (1) those who did not receive MV, (2) those treated exclusively with non-invasive MV, and (3) those that required invasive MV. Patients who used both types of MV could have used non-invasive MV as part of the initial treatment, before intubation, or as part of the MV weaning protocol after endotracheal extubation. Data on the chronology of the use of invasive and non-invasive MV in these patients was unavailable. Thus, patients treated initially with non-invasive MV prior to intubation, or during respiratory weaning, were considered part of the invasive MV group for the purposes of this analysis.

The diagnosis of respiratory failure, the indication for MV, the timing of support initiation, MV weaning strategies and all medical treatment decisions were determined by the medical staff in charge. Non-invasive MV was performed routinely by a respiratory physiotherapist with bi-level positive airway pressure according to standardized procedures. Noninvasive ventilation was delivered by a total face mask, secured with head straps, coupled to a BIPAP Vision^™^ (Respironics INC^®^, Pennsylvania, USA). For patients with a nasogastric tube, a seal connector in the dome of the mask was used to minimize air leakage. After the mask was attached to the patient, pressure support was set at 5 cmH20 to obtain an exhaled tidal volume of 6 mL/kg of predicted body weight, a respiratory rate lower than 30 breaths per minute, attenuation of respiratory accessory muscle activity and achievement of patient’s comfort. Positive end-expiratory pressure (PEEP) was initiated at 10 cm H_2_O and increased in steps of 2 to 3 cm H_2_O up to 15 cm H_2_O until the FiO_2_ requirement was 60% or less. All ventilator settings could be re-adjusted by the attending physician and by a chest physiotherapist, based on the results of continuous oximetry, measurements of arterial blood gases (specially PaCO_2_ and pH) and ventilator parameters (expiratory tidal volume, respiratory rate, and mask leakage) as well as on patients’ comfort. Criteria for endotracheal intubation included failure to maintain an arterial oxygen partial pressure (PaO_2_) > 60 mmHg or SpO_2_ > 90% with an FiO_2_ equal to or greater than 60%, PaCO_2_ higher than 60 mmHg with pH lower than 7.25, inability to protect the airways or to manage copious tracheal secretions, hemodynamic or electrocardiographic instability, inability to tolerate the face mask, inability to correct dyspnea and progression of respiratory failure. Non-invasive MV success patients were maintained coupled to a BIPAP vision continuously during a 24-hour period. Afterwards, parameters were re-adjusted based on SpO_2_, arterial blood gas analysis (specially PaCO_2_ levels), ventilator parameters (expiratory tidal volume, respiratory rate and mask leakage) and patient’s comfort. When FiO_2_ was lower than 50%, respiratory rate lower than 30 breaths per minute, expiratory tidal volume higher than 5 mL/kg of predicted body weight with a pressure support lower than 10 cm H_2_O and PEEP lower than 8 cm H_2_O, non-invasive MV was discontinued and oxygen ventury mask of 50% initiated. If an oxygen ventury mask of 50% was well tolerated during a one-hour period, a ventury mask of 50% was alternated with non-invasive MV (1 hour in ventury mask of 50% and 3 hours in NIV) until the patient could stay spontaneously breathing. The maximal time allowed on full non-invasive MV support was 24 hours. After 24 hours, patients who could not stay for at least one hour on oxygen ventury mask was defined dependent on non-invasive MV and were intubated and mechanically ventilated.

A research nurse team was specifically designated to measure all variables in this registry, including daily reevaluation of data on MV.

The study protocol was conducted in accordance with the Declaration of Helsinki and was approved by the Institutional Review Board of the Hospital Israelita Albert Einstein. The study was granted a waiver for informed consent.

### Statistical analysis

Baseline patient characteristics were summarized by ventilation modality. Continuous variables were expressed as mean ± standard deviation (SD) or medians with interquartile range. Categorical variables were described as absolute and relative frequencies. Analysis of variance or the Kruskal-Wallis test was used to compare numerical variables among ventilation modalities. The chi-square test was used for categorical variable comparisons.

An adjusted Cox proportional hazards regression model was performed to assess the association between both types of MV and in-hospital death, using a stepwise backward selection of variables with entry/stay criteria of p < 0.10/ p < 0.05. Clinical characteristics were also considered to select variables for adjustment. Consequently, fourteen variables were selected by this method: age, gender, diabetes, troponin peak levels, Killip classification on admission, left ventricular ejection fraction (LVEF), body mass index (BMI), STEMI, previous stroke, use of aspirin, thienopyridines, beta-blockers, angiotensin converting enzyme (ACE) inhibitors/angiotensin II receptor blockers (ARBs) and coronary reperfusion for STEMI patients. All statistical tests were two-sided, and the criterion for statistical significance was *P*<0.05. All statistical analyses were performed using SPSS statistical software version 20.0.

## Results

Mechanical ventilation was used in 293 patients (18.2% of the overall population; 54% invasive and 46% non-invasive). Baseline and treatment characteristics of the patients stratified by the presence or modality of MV are described in Tables 1 and 2.

**Table 1:** Baseline characteristics of the patients according to mechanical ventilation treatment.

Characteristics	Pts. without MV (n = 1,317)	Pts. with non-invasive MV (n = 136)	Pts. with invasive MV (n = 157)	*P*
Male (%)	955/1317 (73)	77/136 (57)	113/157 (72)	**0.001**
Pts. without MV vs Pts. with non-invasive MV				**0.001**
Pts. without MV vs Pts. with invasive MV				>0.999
Pts. with non-invasive MV vs Pts. with invasive MV				**0.017**
Age (years), mean ± SD	67±15	78±12	72±14	**<0.001**
Pts. without MV vs Pts. with non-invasive MV				**<0.001**
Pts. without MV vs Pts. with invasive MV				**<0.001**
Pts. with non-invasive MV vs Pts. with invasive MV				**<0.001**
STEMI (%)	490/1237 (40)	41/136 (30)	75/157 (48)	**0.009**
Pts. without MV vs Pts. with non-invasive MV				0.070
Pts. without MV vs Pts. with invasive MV				0.160
Pts. with non-invasive MV vs Pts. with invasive MV				**0.005**
BMI (kg/m^2^), mean ± SD	27±5	27±5	26± 5	0.54
Killip classification (%)				**<0.001**
1	1117/1236 (90.4)	92/136 (67.6)	94/157 (59.9)	
2	81/1236 (6.5)	23/136 (16.9)	24/157 (15.3)	
3	22/1236 (1.8)	17/136 (12.6)	7/157 (4.4)	
4	16/1236 (1.3)	4/136 (2.9)	32/157 (20.4)	
Pts. without MV vs Pts. with non-invasive MV				**0.002**
Pts. without MV vs Pts. with invasive MV				**<0.001**
Pts. with non-invasive MV vs Pts. with invasive MV				0.066
TIMI Risk, STEMI patients	2.90±2.22	4.73±2.67	4.85±2.52	**<0.001**
Pts. without MV vs Pts. with non-invasive MV				**<0.001**
Pts. without MV vs Pts. with invasive MV				**<0.001**
Pts. with non-invasive MV vs Pts. with invasive MV				>0.999
TIMI Risk, Non-STEMI patients	2.55±1.32	3.06±1.34	2.83±1.32	**0.001**
Pts. without MV vs Pts. with non-invasive MV				**0.001**
Pts. without MV vs Pts. with invasive MV				0.207
Pts. with non-invasive MV vs Pts. with invasive MV				0.713
LVEF, mean ± SD	0.54 ± 0.12	0.47 ± 0.13	0.45 ± 0.15	**<0.001**
Pts. without MV vs Pts. with non-invasive MV				**<0.001**
Pts. without MV vs Pts. with invasive MV				**<0.001**
Pts. with non-invasive MV vs Pts. with invasive MV				0.459
Troponin-I (ng/mL),	3.8 (0.58–19.3)	3.2 (0.7–14.0)	7.2 (0.9–2.7)	0.07
median (25th–75th percentile)				
**Coexisting conditions**				
Diabetes mellitus (%)	368/1292 (29)	54/136 (40)	63/156 (40)	**0.001**
Pts. without MV vs Pts. with non-invasive MV				**0.031**
Pts. without MV vs Pts. with invasive MV				**0.012**
Pts. with non-invasive MV vs Pts. with invasive MV				>0.999
Smoking (%)	286/1314 (22)	18/136 (13)	34/157 (22)	0.066
COPD (%)	33/1316 (2.5)	8/136 (5.9)	5/157 (3.2)	0.128
Previous AMI (%)	203/1280 (15.9)	28/132 (21.2)	24/152 (15.8)	0.28
Previous stroke (%)	48/1310 (3.7)	13/136 (9.6)	8/157 (5.1)	**0.005**
Pts. without MV vs Pts. with non-invasive MV				0.066
Pts. without MV vs Pts. with invasive MV				>0.999
Pts. with non-invasive MV vs Pts. with invasive MV				0.439

Data are expressed as mean ± standard deviation (SD), median (25th–75th percentile), or number (%). *P* value was calculated for the comparison among the three groups. MV, mechanical ventilation; BMI, body mass index; STEMI, ST-segment elevation myocardial infarction; LVEF, left ventricular ejection fraction. TIMI, thrombolysis in myocardial infarction.

**Table 2:** Medical treatment and coronary reperfusion according to mechanical ventilation treatment.

Characteristics	Pts. without MV (n = 1,317)	Pts. with non-invasive MV (n = 136)	Pts. with invasive MV (n = 157)	*P*
**Pharmacotherapy during hospitalization**				
Aspirin (%)	1241/1309 (94.8)	124/136 (91.2)	137/157 (87.3)	**<0.001**
Pts. without MV vs Pts. with non-invasive MV				0.444
Pts. without MV vs Pts. with invasive MV				**0.017**
Pts. with non-invasive MV vs Pts. with invasive MV				0.832
Thienopyridine (%)	1069/1289 (82.9)	95/134 (70.9)	88/146 (60.3)	**<0.001**
Pts. without MV vs Pts. with non-invasive MV				**0.009**
Pts. without MV vs Pts. with invasive MV				**<0.001**
Pts. with non-invasive MV vs Pts. with invasive MV				0.179
Beta-blocker (%)	1047/1308 (80,0)	97/136 (71,3)	88/157 (56,1)	**<0.001**
Pts. without MV vs Pts. with non-invasive MV				0.092
Pts. without MV vs Pts. with invasive MV				**<0.001**
Pts. with non-invasive MV vs Pts. with invasive MV				**0.018**
ACE inhibitor or ARB (%)	519/1310 (39.6)	72/136 (52.9)	50/157 (31.8)	**<0.001**
Pts. without MV vs Pts. with non-invasive MV				**0.009**
Pts. without MV vs Pts. with invasive MV				0.148
Pts. with non-invasive MV vs Pts. with invasive MV				0.001
**Reperfusion Therapy**				
Primary PCI or Fibrinolysis/STEMI patients (%)	394/485 (81.2)	28/41 (68.3)	51/75 (68.0)	**0.008**
Pts. without MV vs Pts. with non-invasive MV				0.251
Pts. without MV vs Pts. with invasive MV				0.059
Pts. with non-invasive MV vs Pts. with invasive MV				>0.999
Primary PCI or Fibrinolysis/STEMI pts elegible for reperfusion* (%)	343/369 (93.0)	27/34 (79.4)	44/50 (88.0)	**0.039**
Pts. without MV vs Pts. with non-invasive MV				**0.027**
Pts. without MV vs Pts. with invasive MV				0.665
Pts. with non-invasive MV vs Pts. with invasive MV				0.871

MV, mechanical ventilation; STEMI, ST-segment elevation myocardial infarction; ACE, angiotensin converting enzyme; ARB, angiotensin II receptor blocker. PCI, percutaneous coronary intervention.

*STEMI patients on appropriate time window for reperfusion.

Compared with patients who did not use MV, both groups that used MV were older, more often diabetics, had lower LVEF, higher Killip classification, higher TIMI risk scores and were less treated with thienopyridines or coronary reperfusion. Moreover, the invasive MV subgroup was less treated with aspirin, beta-blockers and ACE inhibitors/ARBs, compared with patients who did not use MV. Compared to exclusively non-invasive MV subgroup, invasive MV patients were younger and more frequently males, had more frequently STEMI, higher rates of patients on Killip 4 classification, a numerically non-significant higher level of troponin and were less treated with beta-blockers and ACE inhibitor/ARBs. Smoking, BMI, previous AMI and COPD rates were similar among the three groups.

The median (25th–75th percentile) lengths of hospital stay were 6 (5.8–6.2), 13 (11.2–4.7), and 28 (18.0–37.9) days, respectively (*P*<0.001). In-hospital mortality rates for patients without MV, with exclusively non-invasive MV, and with invasive MV were 4.0%, 8.8%, and 39.5%, respectively (*P*<0.001).

**Fig 1:**
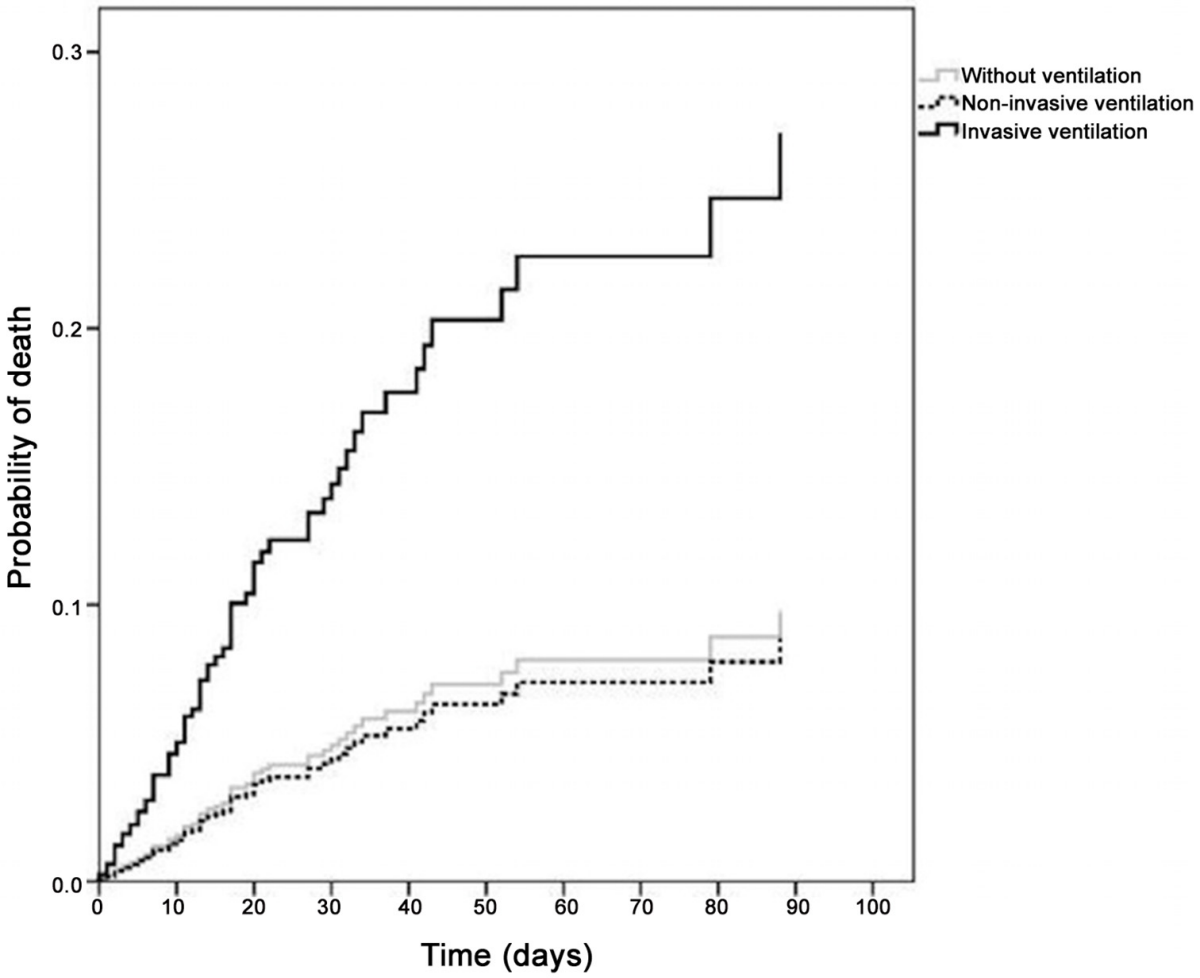
Adjusted mortality curves by ventilation modality.

In the adjusted models (Table 3), compared with the subgroup without MV: (1) non-invasive MV was not associated with in-hospital death (adjusted HR = 0.90, 95% CI 0.40–1.99, *P* = 0.79); and (2) invasive MV was associated with a 3-fold increase in the risk of in-hospital death (adjusted HR = 3.07, 95% CI 1.79–5.26, *P*<0.001). Adjusted survival curves according to ventilation modality demonstrated an increased risk of in-hospital death in patients treated with invasive MV (Fig 1).

**Table 3:** Adjusted Cox proportional hazards regression model.

Variables	HR	95% CI		p
		Lower	Upper	
Age (years)	1.04	1.02	1.06	**<0.001**
Killip classification	1.44	1.18	1.77	**<0.001**
Beta-blocker	0.47	0.29	0.75	**0.001**
ACE inhibitor or ARB	0.25	0.13	0.47	**<0.001**
**Non-STEMI (reference)**				
STEMI patients without coronary reperfusion	2.09	1.13	3.89	**0.019**
STEMI patients with coronary reperfusion	1.10	0.62	1.95	0.736
**Patients without mechanical ventilation (reference)**				
Patients with exclusively non-invasive mechanical ventilation	0.90	0.40	1.99	0.785
Patients with Invasive mechanical ventilation	3.07	1.79	5.26	**<0.001**

ACE, angiotensin converting enzyme; ARB, angiotensin II receptor blocker; HR, hazard ratio. 95% CI, 95% confidence interval; STEMI, acute myocardial infarction with ST-segment elevation. Adjusted for age, gender, diabetes, troponin levels, Killip classification on admission, left ventricular ejection fraction, body mass index, acute myocardial infarction with ST-segment elevation, previous stroke, use of aspirin, thienopyridines, beta-blockers, angiotensin converting enzyme inhibitors/angiotensin II receptor blockers and coronary reperfusion (for patients with acute myocardial infarction with ST-segment elevation).

## Discussion

In our study, we found that among patients with AMI, 18.2% needed treatment with MV. Approximately half of these patients were managed non-invasively. Patients treated exclusively with non-invasive MV had a relatively favorable prognosis, while patients who needed to be treated with invasive MV had a poor prognosis with a three-fold increase in the risk of death.

Patients with AMI may have respiratory failure at presentation, or it may develop during hospitalization due to acute heart failure, non-cardiac pulmonary disorders, or complications of shock. Tsai et al. [1] previously demonstrated that in a STEMI population, 17% were admitted with Killip class III symptoms. Furthermore, nearly 20% of patients who initially presented with Killip class I or II symptoms, ultimately developed more advanced heart failure during their hospitalization. In addition, factors unrelated to myocardial dysfunction may affect respiratory function during MI and, hence, increase the need for MV. Kouraki et al. [4] demonstrated that in 458 AMI patients treated with invasive MV, the initial reason for intubation was non-cardiac in 13%. Further, during hospitalization, 17% of patients developed coma, 14% had severe infections, 10% had sepsis, and 6% showed multiple organ dysfunction syndrome. More recently, Lazzeri et al. [12] evaluated 106 patients with STEMI requiring invasive MV. In this study, the incidence of MV was 7.6%, and the reasons for intubation included cardiogenic shock in 64 patients (60.4%), ventricular fibrillation in 32 patients (30.1%), and acute pulmonary edema in 10 patients (9.5%).

We demonstrated that compared to patients treated without MV, patients treated with invasive or non-invasive MV had a higher Killip class at hospital admission and had lower levels of LVEF. Interestingly, troponin peak levels were relatively modest and similar between groups. This finding suggests that the differences in LVEF levels and heart failure symptoms between groups were not linked with the extension of the acute myocardial necrosis. Additionally, we found that advanced age was also associated with the use of both types of MV. Besides heart failure, aging is particularly related to non-cardiac factors that may increase the risk of respiratory failure, such as dysphagia, delirium, and muscle weakness [13,14]. Recently, Lazerri et al. [12] also observed that patients who received MV were older and more often had had a previous episode of AMI.

Our rate of invasive MV was similar to that previously reported [12]. We demonstrated that AMI patients treated with invasive MV had a poor prognosis with a longer hospital length of stay and high short-term mortality rates reaching almost 40%. Invasive MV was associated with a three-fold increase in the risk of in-hospital death. These data are also similar to previous findings [3,4,12]. Whether this unfavorable prognosis was more related to cardiac or non-cardiac complications (e.g., infections, septic shock, and pulmonary complications of MV) we could not determine. In fact, a cause-and-effect relationship between invasive MV and clinical outcomes could not be established, and our findings might have been influenced by complications of invasive MV such as ventilator-associated pneumonia, delirium and acute respiratory distress syndrome [15,16,17,18]. Importantly, compared to patients treated without MV, the use of cardiovascular pharmacotherapy was less common in the invasive MV subgroup and reperfusion therapy was less common in both, invasive and non-invasive MV subgroups. Whether these differences were related to drug contraindications associated to acute comorbidities (e.g., bleeding, thrombocytopenia, hypotension, acute renal failure and hyperkalemia), delayed admission that may have contraindicated reperfusion on STEMI patients, or presence of type 2 AMI (secondary to ischemic imbalance), we could not determine. Nevertheless, confounders related to cardiovascular pharmacotherapy and coronary reperfusion were included in our adjusted analysis.

On the other hand, clinical and randomized data on AMI patients treated with non-invasive MV are significantly more limited. The efficacy and safety of non-invasive MV in AMI have been tested in randomized small trials with patients who had cardiogenic pulmonary edema of multiple etiologies besides myocardial ischemia. In these trials, compared with standard therapy, non-invasive MV reduced mortality and need for intubation [19–21]. Of note, the effect was more prominent in trials in which myocardial ischemia or infarction was the cause of pulmonary edema in higher proportions of patients [22]. More recently, one small trial has specifically compared non-invasive MV between AMI and non-AMI patients [9]. This study showed comparable benefits in both groups in terms of hemodynamic and respiratory parameters, but was lacking in clinical outcomes data. In our study, we found that almost 10% of the entire population was treated with exclusively non-invasive MV and had relatively favorable outcomes with moderate in-hospital mortality rates. Moreover, exclusively non-invasive MV was not associated with in-hospital death in the adjusted analysis. Our findings suggest that in real-world practice, non-invasive MV is common and may be safe in patients with AMI.

Our study was limited in that it was a retrospective observational single-center study of a relatively modestly sized population. Thus, despite all of the statistical adjustments, one cannot account for the impact of unmeasured confounders on our outcomes. Additionally, we could not adjust our results by global risk scores, such as the APACHE II, by frailty scales, creatinine, albumin or some other prognostic biomarkers since this information was not available in this registry. Nonetheless, besides important clinical and treatment characteristics, we have adjusted our results to troponin levels. This fact further strength our results, since troponin has been considered the most important biomarker in AMI trials and correlates with the extent of myocardial necrosis. It is known that in AMI, other biomarkers such as the B-type natriuretic peptide and C-reactive protein might add prognostic information in selected patients. However, they are not routinely collected in clinical practice, or recommended by guidelines and are not traditionally used in adjusted statistical models in AMI studies

Also, intention to treat analysis was not possible in this study, considering that we have no information on the chronology of the use of invasive and non-invasive MV in those patients that used both strategies. For instance, patients treated with the combination of both types of MV were considered as part of the invasive MV group. This criterion was applied to identify patients who were suitable to be exclusively treated by non-invasive strategies. Thus, we could not identify patients whose clinical status failed to improve after the initial non-invasive treatment and who subsequently needed invasive MV. We also did not have access to data on malignant arrhythmias, defibrillation and cardio-pulmonary resuscitation. Despite the importance of these variables, it is unlikely that adjusting our results for them would change our findings, considering that cardio-pulmonary resuscitation are often associated with other than AMI etiologies of respiratory failure such as pulmonary edema, cardiogenic chock and systemic infections. Lastly, information on the etiology, timing, and medical treatment of the respiratory failure as well as MV duration were not available. Thus, we could not explore mechanisms of respiratory failure and treatment aspects of this group of patients. Consequently, we could not determine whether the high mortality in the invasive MV subgroup was more related to cardiac or non-cardiac complications.

Likewise, we don’t have information about the reasons for lack of coronary reperfusion among some patients in both groups treated with MV. It is possible that a significant part of these patients was not in the appropriate time window for reperfusion. In fact, considering only patients “eligible for reperfusion” (STEMI patients on appropriate time window for reperfusion), reperfusion rates were considerably increased. Nevertheless, both treatment groups had similar rates of reperfusion. Finally, we recognize that our findings do not demonstrate a causality relation between invasive-MV and death. Based on the observational nature of our study, we have only assessed an association between MV and outcomes, and therefore, we could not adjust for unmeasured confounders. Thus, our findings are hypothesis-generating that highlight the need of a large, well-powered, prospective trial comparing the effectiveness of invasive and non-invasive MV in AMI patients with respiratory failure.

In conclusion, in AMI setting, 18% of the patients required MV. Almost half of these patients were treated with exclusively non-invasive MV. This subgroup had a relatively favorable prognosis with moderate rates of short-term death. On the other hand, patients who needed to be treated invasively had poor outcomes and a three-fold increase in the risk of in-hospital death, compared to patients who did not use MV. Thus, future prospective randomized trials are needed to compare the effectiveness of invasive and non-invasive MV for the initial approach of respiratory failure in AMI patients.

## Supporting Information

S1 Dataset Dataset for the entire population. (XLS)

## Abbreviations

AMI: acute myocardial infarction
BMI: body mass index
ECG: electrocardiogram
LVEF: left ventricular ejection fraction
MV: mechanical ventilation
STEMI: ST elevation myocardial infarction
ACE: angiotensin converting enzyme
ARBs: angiotensin II receptor blockers
